# Genome fluctuations in cyanobacteria reflect evolutionary, developmental and adaptive traits

**DOI:** 10.1186/1471-2148-11-187

**Published:** 2011-06-30

**Authors:** John Larsson, Johan AA Nylander, Birgitta Bergman

**Affiliations:** 1 Department of Botany, Stockholm University, SE-106 09, Stockholm, Sweden; 2 Natural History Museum, University of Oslo, P.O. Box 1172 Blindern, NO-0318 Oslo, Norway

## Abstract

**Background:**

Cyanobacteria belong to an ancient group of photosynthetic prokaryotes with pronounced variations in their cellular differentiation strategies, physiological capacities and choice of habitat. Sequencing efforts have shown that genomes within this phylum are equally diverse in terms of size and protein-coding capacity. To increase our understanding of genomic changes in the lineage, the genomes of 58 contemporary cyanobacteria were analysed for shared and unique orthologs.

**Results:**

A total of 404 protein families, present in all cyanobacterial genomes, were identified. Two of these are unique to the phylum, corresponding to an AbrB family transcriptional regulator and a gene that escapes functional annotation although its genomic neighbourhood is conserved among the organisms examined. The evolution of cyanobacterial genome sizes involves a mix of gains and losses in the clade encompassing complex cyanobacteria, while a single event of reduction is evident in a clade dominated by unicellular cyanobacteria. Genome sizes and gene family copy numbers evolve at a higher rate in the former clade, and multi-copy genes were predominant in large genomes. Orthologs unique to cyanobacteria exhibiting specific characteristics, such as filament formation, heterocyst differentiation, diazotrophy and symbiotic competence, were also identified. An ancestral character reconstruction suggests that the most recent common ancestor of cyanobacteria had a genome size of approx. 4.5 Mbp and 1678 to 3291 protein-coding genes, 4%-6% of which are unique to cyanobacteria today.

**Conclusions:**

The different rates of genome-size evolution and multi-copy gene abundance suggest two routes of genome development in the history of cyanobacteria. The expansion strategy is driven by gene-family enlargment and generates a broad adaptive potential; while the genome streamlining strategy imposes adaptations to highly specific niches, also reflected in their different functional capacities. A few genomes display extreme proliferation of non-coding nucleotides which is likely to be the result of initial expansion of genomes/gene copy number to gain adaptive potential, followed by a shift to a life-style in a highly specific niche (e.g. symbiosis). This transition results in redundancy of genes and gene families, leading to an increase in junk DNA and eventually to gene loss. A few orthologs can be correlated with specific phenotypes in cyanobacteria, such as filament formation and symbiotic competence; these constitute exciting exploratory targets.

## Background

Cyanobacteria have played an important role in the history of life on Earth as the inventors of oxygenic photosynthesis, which gradually changed atmospheric chemistry to allow the evolution of Eukarya [1]. Being at the base of global carbon and nitrogen biogeochemical cycles, the latter due to the ability of many genera to fix atmospheric nitrogen gas, has provided them with essential roles in the evolutionary past and in modern ecosystems. The capacity to form stable symbiotic interactions with eukaryotic hosts is another remarkable feature of cyanobacteria, which led to the plastid we today term chloroplasts, and eventually to the plant dominated biosphere of the globe [2,3]. This green plastid is able to capture and transform the light-energy of the sun into biologically useful forms of energy and thereby to fix the carbon that life is built upon. The eukaryotic hosts in contemporary cyanobacterial symbiosis range from the amoeboid *Paulinella chromatophora*, which harbours a unicellular endosymbiotic cyanobacterium [4,5], to a number of plant species spread within the plant kingdom [6]. The latter all act as hosts to the more complex cyanobacteria differentiating for instance heterocysts, a specific cell type in which nitrogen fixation takes place. The frequency of this nitrogen-fixing cell type is often considerably enhanced in symbiosis to support the plant with its total need of combined nitrogen [7].

The flexibility in life styles of cyanobacteria is underpinned by the highly diverse morphology of the cyanobacterial phylum (filamentous/unicellular and multi-cellularity), in their self-sufficiency in terms of physiological capabilities (e.g. photosynthesis and nitrogen fixation), which in turn allows their wide habitat occupancy-range on a global scale (marine/freshwater/soils), often including extreme environments (e.g. from cold arctic to hot springs and desert regions). Cyanobacterial diversity is reflected also at the genomic level. Sequencing efforts over recent years have clearly shown that genomes within the cyanobacterial phylum vary considerably in aspects such as size (~1.4-9.1 Mbp), G+C content (31-63%), number of protein coding genes (1214-8446) and coding nucleotide proportion (52-94%) (see e.g. [8-13]).

We recently reported on the complete genome sequence of the heterocystous cyanobacterial symbiont (cyanobiont), '*Nostoc azollae*' 0708, in the small aquatic fern *Azolla filiculoides *and discovered features signifying a genome in a state of erosion [8]. The *Azolla *symbiosis is a highly integrated mutualistic symbiosis between a pro- and a eukaryote, the heart of which is a nutritional dependence by the plant on the perpetual source of combined nitrogen delivered by the nitrogen-fixing cyanobacterium. This is accomplished through a number of unique features of the *Azolla *symbiosis, many of which suggest a long-lasting co-adaptation between the partners. Firstly, the filamentous cyanobacterium colonizes and is kept as a restricted population in the extracellular cavity that is formed in each *Azolla *leaf [14]. Secondly, the host maintains the cyanobiont population between generations, accomplished through an ingenious and complex vertical transfer mechanism not found in any other plant symbiosis, built on multiple cyanobacterial cell differentiation events [14]. In this process the *Azolla *reproductive organ, the sporocarp, acts as the transfer vehicle of the cyanobacterial inoculum between plant generations. Thirdly, the cyanobiont seems to have lost (at least part of) its autonomy as it can no longer grow outside the plant [15], making it an obligate symbiont, again the only known among plant symbioses.

Interestingly, our genomic analyses showed that the genome of the *Azolla *cyanobiont contains numerous pseudogenes spread over all functional categories. This indicates a severe loss of function, which has forced the cyanobiont to rely on its host for survival. One central question in relation to the cyanobiont of *Azolla *is to what extent the unique obligate life-style of this cyanobacterium has influenced the eroding process of its genome and, more importantly, whether this is a contemporary example of a nitrogen-fixing plant plastid in the making. Recent reports show other interesting examples of reduced genomes within the cyanobacterial phylum, such as the smallest known genomes among the more complex Section IV cyanobacteria (filamentous, heterocystous and plant symbionts), *Cylindrospermopsis raciborskii *CS-505 and *Raphidiopsis brookii *D9 [16] and the streamlined genome of a marine nitrogen-fixing unicellular cyanobacterium (cyanobacterium UCYN-A; [12]). These organisms all show signs of genome reduction, although they, in contrast to the *Azolla *cyanobiont, are not known to form symbioses. *C. raciborskii *differentiates only terminal and *R. brookii *only incompletely developed heterocysts, and the cyanobacterium UCYN-A lacks some genes (among many other present in most cyanobacteria) for photosystem II. Prompted by our findings about the genome of the *Azolla *cyanobiont and these recent reports, combined with the increased wealth of genomic information, we set out to elucidate in greater detail the evolutionary history of the large fluctuations apparent in genome size and content within the cyanobacterial phylum. In the past few years, studies on the phylogeny of cyanobacteria, based upon varying numbers of sequenced genomes and conserved proteins, have been presented [17-19] and aided the identification of major clades within the phylum. The growing number of available genome sequences, however, warrants an updated and robust phylogeny and an analysis of the genomic changes which have occurred within the cyanobacterial lineage.

Based on phylogenetic analysis of 285 orthologous protein groups present in 58 sequenced genomes, we analysed evolutionary patterns of genome composition in cyanobacteria and reconstructed the genome of the most recent common cyanobacterial ancestor. We also assessed the extent of genome reduction and expansion (the former an indication of adaptation to a specific niche, and the latter of a broadened phenotype and adaptation to varying environments) within the phylum and searched for genetic and functional signatures related to morphology, cell development and symbiosis.

## Results

### Phylogenetic analysis and the cyanobacterial core

**Figure 1:**
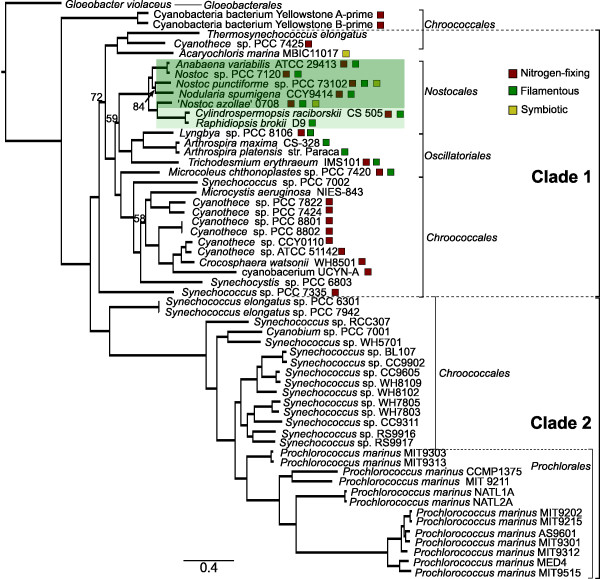
**Phylogeny of 58 cyanobacteria based on a concatenated alignment of core orthologs**. Maximum likelihood phylogenetic tree based on a concatenated alignment of 285 single-copy orthologs present in all genomes. The cyanobacteria with fully developed heterocysts with a regular intercalary pattern distribution are indicated by the dark green shaded box while those with terminal or undeveloped heterocysts are indicated by the light green shaded box. Specific phenotypes are shown by the coloured boxes next to the organism names. Numbers at nodes indicate bootstrap values (when < 100). Bar, 0.4 expected substitutions per site.

Sequences from 58 cyanobacterial genomes were included in this study, representing a mix of unicellular, filamentous and filamentous heterocystous phenotypes, including known plant symbionts, as well as a few potential symbionts. A list of the organisms, organism abbreviations used and general information on their genome is presented in additional file 1. In total, the dataset comprises 217,365 protein sequences, 191,646 (88%) of which were clustered into 16,334 orthologous groups. The phylogenetic relationship of the 58 cyanobacteria was determined based on a concatenated alignment of 285 single-copy orthologs present in all genomes (Figure 1). In the resulting tree, many taxonomic groups are found to be non-monophyletic, for example, the paraphyletic Chroococcales and the polyphyletic Oscillatoriales. On the other hand, the orders Nostocales and Prochlorales are monophyletic. Overall, the major clades are in large agreement with previous studies based on fewer genomes and single genes, such as 16S rRNA or parts thereof [18-21]. The phylogeny can be divided into two main clades, here termed Clade 1 and Clade 2. Clade 1 consists of a wide range and a mixture of phenotypes including both filamentous and unicellular cyanobacteria of the orders Chroococcales (14 genomes), Oscillatoriales (5 genomes) and Nostocales (7 genomes) as well as *Acaryochloris marina *MBIC11017, the latter a unicellular marine organism without an assigned order. All cyanobacteria with known or suspected symbiotic competence belong to Clade 1. The most evolutionary complex representatives within this clade are those capable of cell differentiation, the Nostocales (highlighted in green in Figure 1). The taxonomy of Clade 2 is considerably more uniform, containing only small-celled often closely related marine unicellular cyanobacteria of the orders Chroococcales (15 genomes) and Prochlorales (13 genomes) with small genomes, while the Cyanobacteria bacterium Yellowstone A and B-Prime (also known as *Synechococcus *sp. OS-type A strain JA-3-3Ab and *Synechococcus *sp. strain JA-2-3B'a(2-13), respectively), isolated from hot springs, are placed as a sistergroup to Clades 1 and 2. Eight orthologs were identified in all finished genomes of Clade 1, to the exclusion of all genomes in Clade 2. These orthologs correspond to a techoic acids export protein, a cytoskeleton RodZ putative homolog, a putative multidrug resistance efflux pump, a protein with a membrane protease subunit and four orthologs with unknown function. Conversely, eight orthologs were found in all finished genomes of Clade 2, to the exclusion of all Clade 1 genomes. These correspond to a nuclease, an aminotransferase, a GNAT-family acetyltransferase, a homoserine O-succinyltransferase, a chorismate binding enzyme, the rpoD4 RNA polymerase sigma factor and two orthologs with unknown function. We identified a total of 404 orthologous genes present in all 58 genomes (see additional file 2), a gene set defined here as the most updated cyanobacterial "core". The most abundant functional protein categories encoded by this strict core were not unexpectedly those related to translation (80 orthologs), but categories such as coenzyme metabolism (39 orthologs), DNA replication, recombination and repair (35 orthologs), energy production (33 orthologs) and cell membrane biogenesis (29 orthologs) were also highly represented. Four genomes had lost functional gene copies in five of the 404 core orthologous groups (discussed further below). By defining a relaxed core gene set, based on orthologs present in the 39 finished genomes (with both presence and absence allowed in unfinished genomes), we identified 132 additional orthologs, of which 103 are single-copy genes (see additional file 2). This relaxed core, termed RCF, contained 23 orthologs involved in translation, 11 of which are ribosomal proteins and the rest are tRNA synthetases, elongation and release factors and one uncharacterised gene putatively involved in translation repression. Within RCF are also several DNA maintenance genes such as *recA*, *recG *and *mutS*, genes involved in carbohydrate and amino acid metabolism such as glucose-6-phosphate dehydrogenase and glutamine synthetase as well as some subunits of the ATP synthase and photosystem I. A further relaxation of the core, obtained by allowing orthologs to be missing from one (RCF1), two (RCF2) and three (RCF3) finished genomes, resulted in an additional 268, 86 and 57 identified gene families, respectively (see additional file 2). In all of these relaxed cores, the highly reduced genome of cyanobacterium UCYN-A (Ucyn) consistently lacked the highest proportion of orthologs with 179, 44 and 32 genes missing from RCF1, RCF2 and RCF3, respectively. Several orthologs were also missing from the deep branching species *Gloeobacter violaceus *(Glov) and Cyanobacteria Yellowstone A- and B-prime (YellA and YellB). Glov lacked 36 orthologs in RCF1: e.g. the circadian clock kinase gene *kaiC*, genes involved in transport of cobalt, iron and copper as well as several genes of unknown function. YellA and YellB both lacked 11 orthologs in RCF2: e.g. the gamma subunit of the NAD hydrogenase (*hoxS*), an N-acetyltransferase involved in membrane biogenesis, a putative hydrolase and several uncharacterised genes.

In total, 18 finished genomes were missing genes, or had only pseudogenes, in one or more orthologous groups where all other genomes were represented (see additional file 3). These missing orthologs are likely the result of specific adaptations for each individual genome, or of extensive divergence of proteins that may cause them to escape identification. The marine unicellular cyanobacterium UCYN A (Ucyn), misses 127 orthologs present in all other genomes corresponding to genes such as those coding for proteins of the PSII complex (see additional file 3). Its minimal genome (~1.44 Mbp) and lack of crucial genes suggest that Ucyn is in reality an obligate symbiont, although no eukaryotic host has been found [22]. In most other genomes only a few (1-19, maximum in Glov) orthologs were missing or non-functional.

'*Nostoc azollae*' 0708 (NoAz) is the only obligate plant symbiont among the 58 cyanobacteria investigated; the reason for its obligate dependence on its host is as yet unknown. However, we identified two protein groups present in all other genomes, which in NoAz were non-functional pseudogenes. These correspond to a geranylgeranyl pyrophosphate synthase and uroporphyrinogen-III synthase HemD. The former is involved in the synthesis of carotenoids and chlorophyll, and the latter is generating precursors of tetrapyrroles such as haem, chlorophyll and bilins, all with important roles in photosynthesis and protection against photooxidative damage.

Of all the protein groups identified, 5127 (31%) are unique to cyanobacteria. That is, they contain no sequences with similarity to proteins in organisms outside the cyanobacterial phylum (BLASTP e-value < 0.01, nr database 24 June 2010). Only two of these unique protein groups, which we term Cya1043 and Cya1555, are represented in all 58 genomes (corresponding to genes *all2080 *and *all0476 *in *Nostoc *sp. PCC 7120, respectively). Cya1043 corresponds to a AbrB family transcriptional regulator, shown to be involved in triggering a number of physiological processes in cyanobacteria, such as nitrogen metabolism [23], toxin production [24], photosynthesis [25] and oxidative stress [26]. Paralogs of this gene are present in 13 genomes, with the highest total number of gene copies in *Acaryochloris marina *(11 copies in total, three of which are present on the main chromosome). Although no known function could be ascribed to Cya1555, its relative organization in the genomes was highly conserved. In 53 of the 58 genomes the gene was found directly upstream of *murG*, involved in cell wall and membrane biogenesis. The genomes that represented exceptions to this gene organization were those of the five unicellular strains *Synechocystis *sp. PCC 6803 (Scys6803), *Thermosynechococcus elongatus *(Thee), *Gloeobacter violaceus *(Glov) and Cyanobacteria bacterium Yellowstone A- and B-prime (YellA and YellB). In YellA and YellB the Cya1555 gene is situated between a rhodanese domain protein and an isoleucyl-tRNA synthetase. In Thee, a three-gene cluster composed of the *ctaCDE *cytochrome c oxidase subunits is located upstream of the gene. Lowering the cut-off on searches (at e-value 0.045, Uniprot database) we found that Cya1555 is distantly related to a DNA-directed RNA polymerase of *Mycoplasma penetrans*. Among the relaxed core sets, an additional seven orthologs are unique to cyanobacteria. One of these orthologs contains a thioredoxin domain (the gene is missing from the *Prochlorochoccus *NATL1A and NATL2A genomes) while the other cyano-unique orthologs in the relaxed core sets corresponded to proteins of unknown function.

### Gene duplications

**Figure 2:**
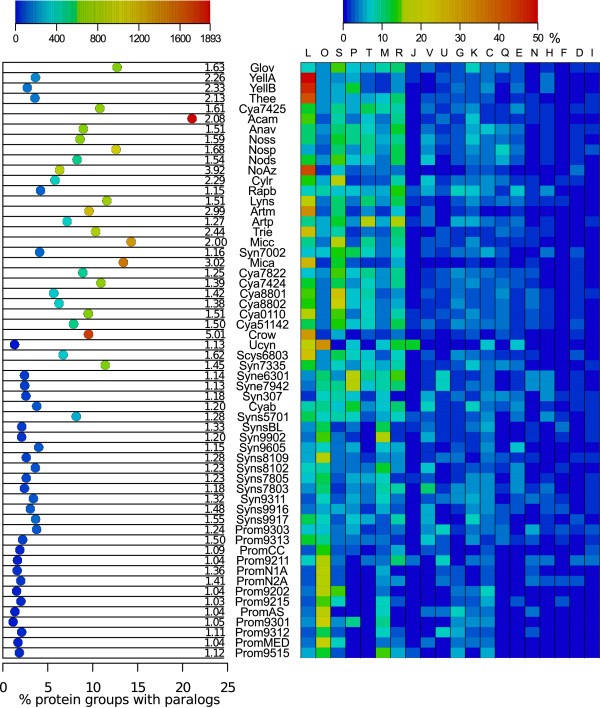
**Amount and functional distribution of paralogs in cyanobacterial genomes**. To the left a plot of proportion of protein groups containing paralogs (x-axis) and total number of paralogous gene copies (colour of circles). Numbers in the right margin of the plot show the average number of gene copies for paralogs. Organisms are ordered according to their position in the phylogenetic tree. To the right a heat map of the functional distribution of paralogs classified according to the Cluster of orthologous groups (COG) categories. Abbreviations for functional categories are as follows: t, a subset of L containing 26 transposase categories; O, posttranslational modification, protein turnover and chaperones; S, function unknown; L, replication, recombination and repair; P, inorganic ion transport and metabolism; T, signal transduction mechanisms; M, cell wall/membrane/envelope biogenesis; R, general function prediction only; J, translation, ribosomal structure and biogenesis; V, defence mechanisms; U, intracellular trafficking, secretion and vesicular transport; G, carbohydrate transport and metabolism; K, transcription; C, energy production and conversion; Q, secondary metabolites biosynthesis, transport and catabolism; E, amino acid transport and metabolism; N, cell motility; H, coenzyme transport and metabolism; F, nucleotide transport and metabolism; D, cell cycle control, cell division and chromosome partitioning; I, lipid transport and metabolism. Categories are ordered by maximum percentage.

Of the total 16,334 orthologous protein groups identified, 5216 contained recent paralogs (in-paralogs) in one or more genome, 1642 of which contain only in-paralogs from a single species lineage, arising from duplication after speciation or representing ancient duplications retained in one lineage only. We made no attempt to discriminate between the origin of paralogs (i.e. horizontal gene transfer or intra-chromosomal duplication events). However, as the clustering method used (OrthoMCL, see Methods) identifies genes within genomes that are more similar to each other than to genes in other genomes our analysis of paralogs consequently refers to in-paralogs. The highest gene-copy number for any single protein group was found for a transposase sequence, with for instance 292 copies in *Crocosphaera watsonii *(Crow). We determined the proportion of paralogs and the total number of paralogous gene copies in each genome and classified these according to COG functional categories (Figure 2). Figure 2 (left) clearly shows that large genomes of Clade 1 dominate the proportion of paralogs as well as the total paralogous gene copy number in our dataset. The genomes with the highest number in both these categories is the unicellular *Acaryochloris marina *(Acam), in line with previous reports [9,27], but high numbers are also encountered in *Microcystis aeruginosa *(Mica) and *Crocosphaera watsonii *(Crow), the filamentous *Microcoleus chthonoplastes *(Micc) and the heterocystous *Nostoc punctiforme *(Nosp). It is interesting to note that both Acam and Nosp represent cyanobacteria with symbiotic competence (see Discussion below). The average number of gene copies for paralogs is highest in Crow (an average of five copies per paralog). We found that most paralogs cannot be classified according to COG functional categories (not shown in Figure 2) and that the majority of genomes in Clade 1 have a higher distribution of paralogs in the Replication (L) and Signal transduction (T) categories, compared to Clade 2 (Figure 2, right). The high abundance in the latter category indicates that Clade 1 cyanobacteria have focused protein family expansions towards sensory functions, thus increasing their capacity to adapt to varying environmental conditions. The numerous paralogs in the replication category are mostly linked to transposases which can be seen in Figure 2 where the "t" column is a sub-set of the L category, representing 26 transposase COGs. Transposases are genetic elements that may also increase adaptation in bacteria [28-30]. Additionally, the poorly characterized categories S and R are more abundant among paralogs in Clade 1, indicating putative novel inventions in these organisms. In contrast, paralogs in Clade 2 have a higher distribution in the Posttranslational modification and chaperone (O) and the Cell membrane biogenesis (M) categories, suggesting an increased need for maintaining correct protein folding and high membrane stability during rapid growth. Ucyn, which has the smallest genome not only within Clade 1 but among all 58 cyanobacteria in this study, also contained the lowest number of paralogs. Therefore, the few examples of paralogs found within the Ucyn genome are highly interesting. These show a high distribution in the Replication (L), Post translational modification and chaperone functions (O), Inorganic ion metabolism (P), Cell membrane biogenesis (M), Translation (J), Energy production (C) and Amino acid metabolism (E) categories. The obligate plant symbiont '*Nostoc azollae*' 0708 (NoAz) is unusual in that it contains comparatively few paralogs, most of which fall into the Replication (L) category, and to some degree the Cell cycle control, cell division and chromosome partitioning (D) functional classes, and the uncharacterised S category. A most striking feature is that not less than 725 of the 1136 (64%) paralogs are pseudogenes in NoAz as are 252 of the 1202 (21%) in *Trichodesmium erythraeum *(Trie).

### Evolutionary history of genomes: size and composition

**Figure 3:**
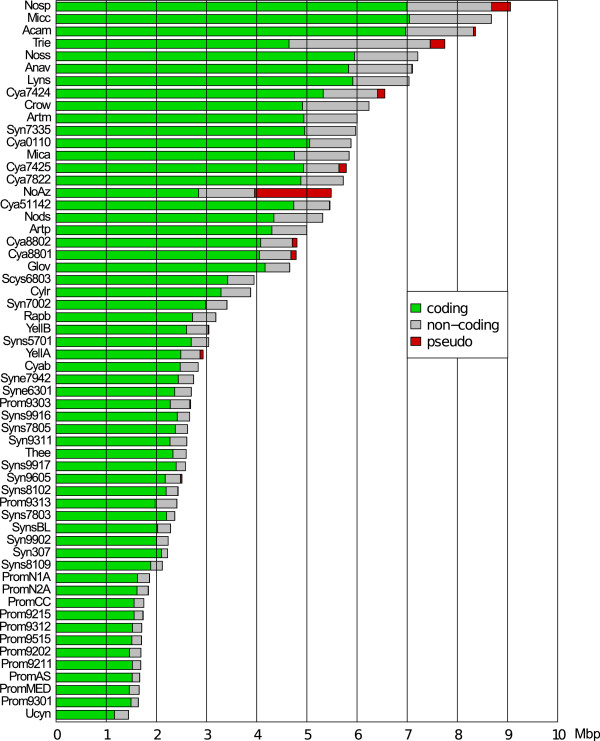
**Genome sizes among 58 cyanobacteria**. The bars denote the genome size in Mbp and the green, grey and red portions of the bars represent nucleotides in coding, non-coding and pseudogene regions, respectively.

The genome size variation among the 58 cyanobacteria examined, illustrated in Figure 3, is formidable, spanning from 1.4 (Ucyn) to 9.3 Mbp (Nosp). Cyanobacteria with a genome size of > 3.3 Mbp (Rapb and above) are more variable in terms of cell size and include all filamentous cyanobacteria, while all genomes < 3.3 Mbp in size (YellB and below) represent unicellular cyanobacteria, restricted to oceans or hot springs. Cyanobacteria with larger genomes are also considerably more variable in their habitat selection, occupying soils, limnic and marine environments as well as a range of eukaryotic symbiont hosts (see additional file 1). Within this group there is also a large subclade of unicellular cyanobacteria with larger genomes, represented by Crow, Mica and the six genomes within the genus *Cyanothece*. It is also obvious that cell differentiation is not related to genome size as the heterocystous cyanobacteria represent the largest (Nosp) and the smallest (Rapb) genomes within the > 3 Mbp genome group. However, the two cyanobacteria with smallest genomes in the heterocystous clade (Figure 1), Rapb, with 'traces' of heterocysts, and Cylr, with terminal heterocysts only, may be on their way to lose the cell differentiation machinery [16]. Indeed, gene loss is probably also the case for Ucyn which, in spite of belonging to Clade 1 cyanobacteria (Figure 1), has the smallest genome of all 58 analysed here. While coding nucleotides (green parts of the bars in Figure 3) occupy most of the cyanobacterial genomes, those of Trie and NoAz differ considerably in having a significant proportion of their genomes represented by non-coding DNA and a high frequency of pseudogenes, respectively (Figure 3). A Pearson's chi-squared test based on 2000 replicates show that non-coding nucleotides are unequally distributed in cyanobacterial genomes (p-value < 0.001) and that NoAz and Trie are heavily biased in this respect (data not shown). Another 13 genomes show a positive bias in non-coding nucleotides, most of which are in the larger range of genome sizes (Figure 3; Nosp-Anav, Cya7424-Syn7335, Mica and Nods), while the majority of genomes < 3 Mbp are biased towards coding nucleotides. The organisms *Prochlorococcus *MIT 9313 (Prom9313) and Ucyn harbour the only genomes < 3 Mbp which display an over-representation of non-coding nucleotides.

**Figure 4:**
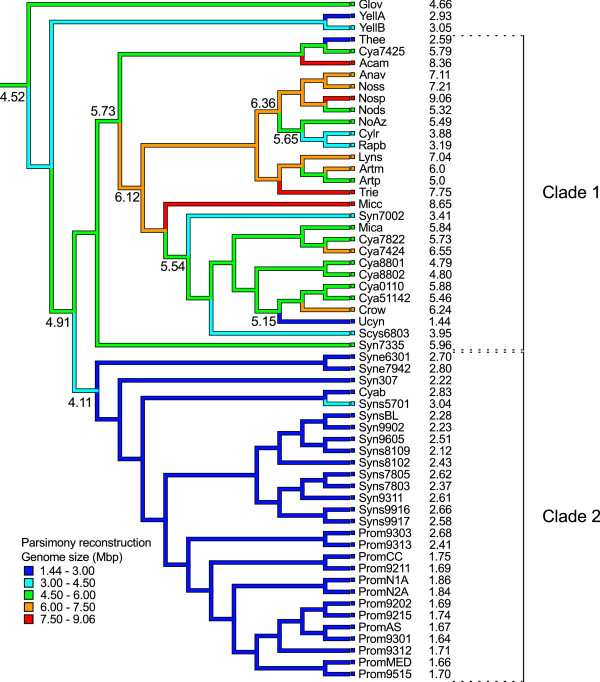
**Parsimony reconstruction of ancestral genome sizes**. Genome sizes are indicated (in Mbp) at specific nodes and in the right margin for the extant genomes. A single event of genome reduction appears to have occurred in the ancestor of the unicellular and mostly marine cyanobacteria (*Prochlorococcus*/*Synechococcus*). In contrast, the history of Clade 1 cyanobacteria involves a mix of genome expansions and reductions. The most notable expansion events are evident in the genome of *Acaryochloris marina *(Acam), *Trichodesmium erythraeum *(Trie), *Nostoc punctiforme *PCC 73102 (Nosp) and *Microcoleus chthonoplastes *(Micc). Reductions in this clade include *Cylindrospermopsis raciborskii *(Cylr), *Raphidiopsis brookii *(Rapb), *Synechococcus *sp. PCC 7002 (Syn7002), *Synechocystis *sp. PCC 6803 (Scys6803) and cyanobacterium UCYN-A (Ucyn). Note that the genome size of the obligate symbiont '*Nostoc azollae*' 0708 (NoAz) has not changed considerably since the ancestor shared with Cylr and Rapb although the pseudogenization in NoAz is considerable (Figure 3).

To visualize the fluctuations of genome sizes within the cyanobacterial lineage we applied a parsimony reconstruction of genome size at the nodes of the phylogeny. As seen in Figure 4, expansions have primarily occurred within Clade 1 cyanobacteria, and a few genomes within this clade appear to have undergone particularly significant genome expansions: Acam, Trie and Micc. Among the seven heterocystous cyanobacteria, the genome of Nosp has expanded to become the largest known for cyanobacteria today, while the genomes of *Cylindrospermopsis raciborskii *(Cylr) and *Raphidiopsis brookii *(Rapb) have undergone severe reductions. In the subclade consisting of these two cyanobacteria and NoAz, a decrease in genome size has occurred since the branching from the *Anabaena*/*Nostoc *clade. However, whereas NoAz has retained roughly the size of the ancestor shared with Cylr and Rapb, the reductions in these latter genomes have been more severe. Genome sizes have decreased also in *Synechococcus *sp. PCC 7002 (Syn7002) and *Synechocystis *sp. PCC 6803 (Scys6803), while the other members of this subclade are dominated by the more large-celled unicellular cyanobacteria of the genus *Cyanothece*, in which no obvious size changes appear to have occurred since their branching from Micc. However, the most notable genome reductions have occurred in Ucyn and *Thermosynechococcus elongatus *(Thee). In sharp contrast to the high levels of genome size fluctuations of Clade 1 cyanobacteria, a single event of genome reduction appears to have taken place in Clade 2 cyanobacteria, from the original 4.11 Mbp to the less than 3 Mbp genome sizes (Figure 4). The size of the genome at the branching point between Clade 1 and Clade 2 cyanobacteria is in turn estimated to be 4.91 Mbp.

**Figure 5:**
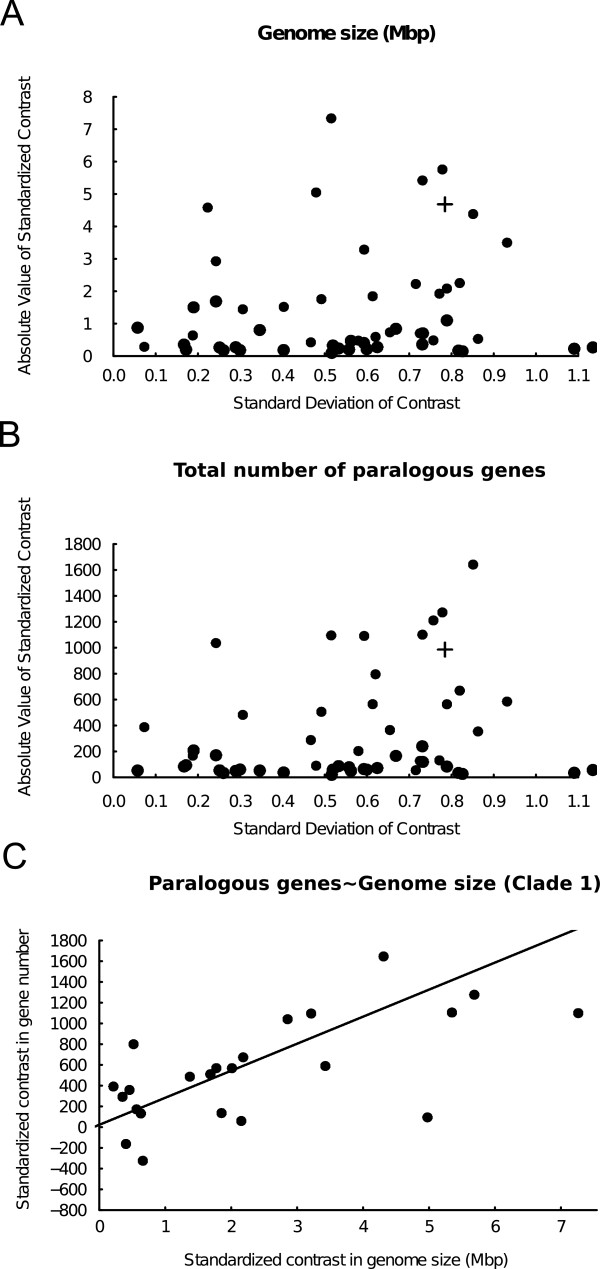
**Relationships between genome sizes and paralog number**. A and B) Diagnostic plots of absolute values of standardized independent contrasts in genome size (A) and number of paralogs (B) versus their standard deviations (square roots of sums of branch lengths) for Clade 1 (closed circles) and Clade 2 (open circles) cyanobacteria. The plots shows that contrasts within Clade 1 are, on average, larger in magnitude (Wilcoxon tests, two-tailed p < 0.0001). This indicates that genome sizes and gene-copy numbers have evolved at a higher rate in Clade 1 species. In A and B, a plus sign denotes the contrast between Clade 1 and Clade 2, which was excluded from the tests. C) Phylogenetically correct regression analysis through the origin of contrasts in number of paralogous gene copies vs. positivized contrasts in genome size for Clade 1. The plots show a strong positive correlation between genome size and number of duplicated genes (r = 0.691, two-tailed p < 0.0001).

Differences in genome sizes between Clade 1 and Clade 2 were analysed using Felsenstein's phylogenetically independent contrasts [31,32]. As seen in Figure 5A,B, Clade 1 cyanobacteria (filled symbols) show a higher evolutionary rate of both genome size (Wilcoxon-test, p < 0.0001) and paralog copy-number (same test, p < 0.0001). As the differences between the clades make them unsuited for simultaneous regression analysis through the origin [33], subsequent analysis was focused on the more heterogeneous cyanobacterial genomes of Clade 1. A phylogenetically correct regression analysis of paralogous gene copy number (dependent variable) and genome size (independent variable) within Clade 1 shows a significant positive correlation between genome size and number of duplicated genes (r = 0.691, two-tailed p < 0.0001) (Figure 5C). This is in line with previous reports [27,34,35]. We also noted a negative correlation (r = -0.252) between genome size and coding nucleotide proportion within Clade 1, although without statistical support (two-tailed p = 0.205).

As seen in Figure 4, the inferred genome size of the most recent common ancestor (MRCA) of cyanobacteria is approximately 4.5 Mbp. Based on the presence/absence of orthologs in the nodes of the tree, the genome of the MRCA is estimated to have contained between 1678 and 3291 protein-coding genes (see additional file 4). Given the large number of paralogs in the genomes at the tree nodes we also determined the gene content of the MRCA based on the copy number of genes in orthologous groups. However, this did not shift the gene content to a large extent (1816-3570 protein-coding genes). The coding nucleotide proportion of the MRCA, based on the lowest and highest predicted number of genes is 36 and 70%, respectively. This indicates that the smaller size of the gene set is likely to be an underestimate. Of the estimated MRCA gene set, 206 of the maximum 3291 and 68 of the minimum 1678 orthologs are unique to cyanobacteria. In total, 90 of these cyano-unique groups could be reliably annotated in one or more of the databases used (see Methods). The majority of cyano-unique orthologs in the MRCA gene set to which a COG category could be assigned belonged to the categories Function unknown or General function prediction only (12 orthologs), Transcription (4 orthologs), Replication, recombination and repair (4 orthologs) and Posttranslational modification, protein turnover, chaperones (4 orthologs). Furthermore, this unique set of ancestral cyanobacterial proteins includes an uncharacterised thylakoid-associated protein, a thylakoid membrane protein, the replication initiation and membrane attachment protein (DnaB), four bacterial conjugation TrbI-like proteins, a number of phycocyanin-associated proteins and the circadian clock protein KaiA. Additionally, the MRCA gene set includes the genes *nifEHDKUB*, involved in the nitrogen fixation process, and the transcriptional regulator *patB*. However, the presence of these genes in the MRCA could not be inferred unambiguously.

### Phenotypic characteristics and functional distribution

**Figure 6:**
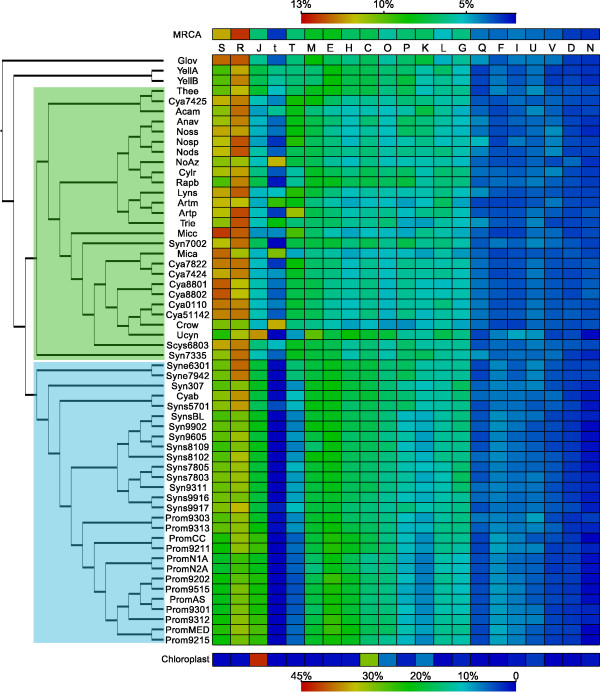
**Functional distribution of genes in the cyanobacterial genomes**. The distribution of functions for the most recent common ancestor (MRCA) of cyanobacteria is shown at the top. Clade 1 and Clade 2 cyanobacteria are shaded in green and blue in the phylogeny, respectively. At the bottom of the plot is the distribution for chloroplasts (averaged over 189 genomes with a total of 17,216 genes). Note that the chloroplast plot has its own colour key (bottom). COG categories are ordered according to maximum contribution in a single genome. Some Clade 1 cyanobacteria appear to have a higher distribution of genes related to Replication (L), Signal transduction mechanisms (T) and Cell motility (N), while functions related to Amino acid metabolism (E) and Posttranslational modification and chaperones (O) are more abundant in Clade 2. Pseudogenes have been excluded from this analysis. COG abbreviations are as in Figure 2.

An overview of phenotypic characteristics represented by the 58 cyanobacteria examined is given in additional file 1: morphology (filamentous, unicellular and heterocyst differentiation), physiology (nitrogen-fixation), symbiotic competence, and typical habitat (marine, freshwater, terrestrial and hot springs). To search for any biased functional distribution, which may indicate functions necessary for a specific phenotype/characteristic, COG functional categories were attributed to each protein in the 58 genomes. The relative distribution of COG categories is shown in Figure 6 in relation to their phylogenetic position. The functional distribution of genes in chloroplast genomes - an extreme case of cyanobacterial genome reduction and adaptation - and in the predicted gene set of the cyanobacterial MRCA is also given. Some conspicuous trends are apparent. For instance, the category Replication, recombination and repair (L) makes up 10%, or more, of the gene repertoire in five genomes: Crow, NoAz, Mica, Artm and Trie. Most of these genes are related to transposases in each genome as can be seen in Figure 6 where orthologs present in 26 transposase COGs in the L category are displayed separately ("t" column). In the majority of other genomes, the L category contributes 5% or less and transposases are scarce in Clade 2 genomes. In NoAz, most genes in the L category (including transposases) are pseudogenes, but several functional transposases still exist in the genome (Figure 6). The two other COG categories with the highest proportion of genes in most genomes are General function prediction only (R), dominating in all genomes including the MRCA, and Function unknown (S), indicating that a large part of the proteins in cyanobacteria are as yet not classified. The latter of these two categories was generally more abundant in genomes belonging to Clade 1 cyanobacteria. The smaller cyanobacteria of Clade 2 more consistently contain a higher proportion of genes related to amino acid metabolism (E) but a lower proportion of genes involved in signal transduction (T) and cell motility (N) compared to most other cyanobacteria. Furthermore, the minimal genome of Ucyn has 10.5% of its genes in the translation (J) category, and its functional bias is thereby similar to that in chloroplasts, but not to its closest relatives. The extremely reduced genomes of chloroplasts are also highly devoted to energy production and conversion (C), while Ucyn has a low proportion of its genes in this category. Rather this genome appears biased towards cell membrane biogenesis (M) and coenzyme transport and metabolism (H). It should also be noted that the functional distribution of the MRCA is not particularly similar to that of chloroplasts, although it may have been closely related to the chloroplast progenitor.

**Figure 7:**
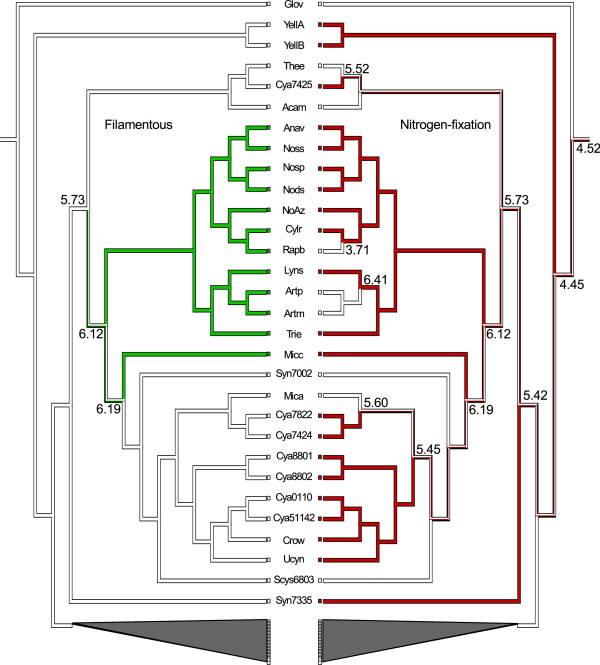
**Parsimony reconstruction of ancestral states of the filamentous and nitrogen-fixing phenotypes**. Equivocal inference are indicated with bi-coloured branches. Genome sizes (in Mbp) is indicated at key nodes of loss/gain. Both characters show a homoplastic pattern with a possible single common origin of the trait followed by one (A) or many (B) losses.

The filamentous phenotype is exhibited by 12 organisms in our dataset and shows an ambiguous evolutionary history as seen in Figure 7A. Parsimony reconstruction cannot discriminate between the possibilities ranging from two independent gains (in Micc, and the Anav-Trie clade, respectively) to one common gain (in the large sister group to Thee/Cya7425/Acam) with a consecutive loss of the trait (in the ancestor of Syn7002-Scys6803). The result of this reconstruction is in line with previous studies of cyanobacterial traits [36,37]. We found 25 orthologs (see additional file 5) present in all filamentous cyanobacteria with genomes sequenced to completion, but lacking in all unicellular cyanobacteria. Neither of these orthologs were present in the predicted gene set of the cyanobacterial MRCA but nine are unique to the cyanobacterial phylum. Notably, eight of the 25 orthologs are pseudogenes in NoAz, which implies that they are not directly involved in filament formation. Of the remaining 17 orthologs, two are present in all 12 filamentous cyanobacteria and correspond to a thermonuclease (Pfam ID PF00565) and a protein distantly related to a bacterial regulator of the *luxR *family (Pfam ID PF00196 at e-value = 0.18). These two orthologs were identified previously by Stucken and co-workers [16] in a set of genes found only in filamentous cyanobacteria. However, the former of the two was not included in their proposed core set (present in all filamentous species) comprising 10 genes in total. Of the remaining 15 orthologs found only in filamentous cyanobacteria, six were also identified by Stucken and co-workers. Six of the additional nine orthologs identified here show significant similarity to a sucrose porin, a protein kinase, a putative anti-sigma factor antagonist (involved in sporulation in *Bacillus subtilis *[38]), a peptidase-like protein, a protein with a xylose isomerase domain and a protein with a KGK domain (Pfam ID PF08872). The two latter are unique to cyanobacteria. Three of the nine orthologs show only slight similarity to a histidine kinase, an outer membrane efflux protein and a protein involved in fatty acid synthesis (Pfam IDs PF00672, PF02321 and PF00550 at e-values 0.024, 0.066 and 2.000, respectively). We found no orthologs uniquely shared by unicellular cyanobacteria, which provides additional support to the hypothesis that the filamentous phenotype is a derived trait [16].

Of the 58 cyanobacteria in the dataset, 21 fix atmospheric nitrogen (diazotrophs). The history of nitrogen fixation, as explained by parsimony and our taxonomic sample (Figure 7B), does not exclude the possibility that the cyanobacterial MRCA was diazotrophic. A number of gains and losses of this capacity have clearly occurred throughout the evolution of the cyanobacterial lineage. Nitrogen fixation is, as expected, present in all heterocystous cyanobacteria with the notable exception of Rapb, an example of an organism in the process of losing capacity to differentiate heterocysts and diazotrophy [16]. Another example of loss is seen in the genus Arthrospira (earlier Spirulina). Interestingly, diazotrophy is retained in a number of unicellular cyanobacteria outside Clade 2, including Ucyn with its minimal genome, while it seems to have been lost in the globally wide-spread Mica and in Syn7002 and Scys6803. Hence, there is a general positive correlation between diazotrophy and filamentation on one hand and unicellular cyanobacteria with larger genomes on the other, while the smaller Clade 2 cyanobacteria lack this capacity. The *hetR *gene, encoding the master regulator for heterocyst development [39], is also not unexpectedly lacking in Clade 2. The presence of *hetR *shows a positive correlation with diazotrophy (being present in 10 out of the 21 diazotrophs). The correlation is even stronger between *hetR *and the filamentous phenotype, whether heterocystous or non-heterocystous, with *hetR *being present in all 12 filamentous phenotypes examined here. The *hetR *gene is in addition found in two unicellular strains of *Synechococcus *(7335 and 7002), being a diazotroph and a non-diazotroph, respectively, where its function is unknown.

Thirteen orthologs were found in all nitrogen-fixing cyanobacteria with finished genomes, but lacking in all non-diazotrophs. Six of these are present in all nitrogen-fixing species and correspond to five genes in the nitrogenase (*nif*) gene cluster: *nifBEHKS *(the *nifD *gene is not included in this ortholog set because its amino acid sequence was missing from the NCBI protein fasta file of *Nostoc punctiforme *although the gene exists in this genome with a DNA incision element). The remaining ortholog is the XRE family transcriptional regulator *patB*, which is found in the vicinity of the *nif *cluster in a few cyanobacteria, but is distantly located in others. Rapb, which appears to have lost the complete *nif *cluster [16], did not share any orthologs exclusively with all nitrogen-fixers or all nitrogen-fixers with finished genomes. Similarly to the flanking regions of the *nif *cluster in Cylr, Rapb shows a high similarity to both sides of the *patB *gene in Cylr, suggesting a location for *patB *in Rapb before being lost. The additional six orthologs found only in nitrogen-fixing cyanobacteria correspond to Ferredoxin-3 and the *nifUWXZT *gene cluster.

Heterocysts are formed by seven organisms in our dataset. We identified 96 orthologs present in all heterocystous cyanobacteria with finished genomes that were absent from all non-heterocystous cyanobacteria (see additional file 6). As for the filamentous phenotype, neither of the heterocyst signature genes were present in the predicted cyanobacterial MRCA. However, 41 of the 96 heterocyst specific orthologs were unique to cyanobacteria. Among these, which mostly correspond to hypothetical genes, are two genes involved in bacterial conjugation (TrbI-like proteins), a gene with a domain found in osmotic shock protection proteins (Pfam ID PF04972) as well as a putative type IV pilus biogenesis gene. Two of the cyanobacteria included in the study do not exhibit a complete developmental mode. Cylr forms only terminal heterocysts and Rapb only partially developed terminal heterocysts [16]. The response regulator gene *patA*, known to be essential to proper heterocyst pattern formation [40,41], is among the 96 heterocyst specific orthologs. Rapb lacks this ortholog and a protein alignment of PatA shows that the conserved N-terminal PATAN domain [42] of the protein is missing in Cylr, which may underpin the presence of exclusively terminal heterocysts in this organism. Of the 96 heterocyst-specific orthologs, not less than 21 orthologs are pseudogenes in NoAz, most of which are involved in solute transport and cell membrane biogenesis. One of the orthologs present in all heterocystous cyanobacteria was found in 3 to 10 copies in all genomes except in NoAz where it is a single-copy gene. No function could be ascribed to this ortholog with the cut-off used. However, lowering the cut-off (at e-value 0.087 in the Pfam database) we find that the ortholog is distantly related to fimbrial proteins (Pfam ID PF00419), which enable bacteria to colonize the epithelia of host organisms and to promote virulence [43].

The only two proven plant symbionts examined in this study, Nosp and NoAz, exclusively share 13 orthologs (see additional file 7). Notably, NoAz has lost function in seven of these, two belonging to the receptor/sensory family of proteins, one glycosyl transferase involved in cell membrane biogenesis, a threonine/homoserine efflux transporter, a type I phosphodiesterase, a short-chain dehydrogenase of unknown specificity and one ortholog to which no function could be assigned. Of the remaining six orthologs exclusive to the plant symbionts, functions could be assigned to two orthologs: a signal transduction histidine kinase and a glycosyl hydrolase (Pfam ID PF01374) which catalyses the endohydrolysis between N-acetyl-D-glucosamine and D-glucosamine. No other genome in the dataset contained any proteins annotated with this Pfam ID. Two orthologs were exclusive to NoAz, Nosp and Acam, the latter obtained as a symbiont in colonial ascidians [44], one of which corresponds to a ADP-ribosylglycohydrolase while to the other no function could be ascribed. Additionally, three orthologs were exclusive to NoAz, Nosp, Cylr and Rapb, which may be forming associations. These correspond to a protein with an S-layer homology domain (which coat the surface of bacteria), a Tellurite-resistance protein and a protein with unknown function.

In contrast to Nosp, which is a facultative symbiont, NoAz spends its entire life cycle in a perpetual plant interaction and has suffered severe loss of function with a massive number of pseudogenes mainly in the functional categories related to replication, secondary metabolite biosynthesis, and signal transduction [8]. We reconstructed the ortholog content of the most recent ancestor of NoAz and its closest relatives, Cylr and Rapb. Assuming that NoAz entered into its obligate symbiotic relationship (potentially 140 million years ago [45]) after diverging from Cylr and Rapb, this represents the last known free-living ancestor of NoAz. Thus, any orthologs in this subset which are missing in the extant NoAz genome should have been lost during its evolution within the plant environment, and possibly reflect redundant functions in this evolutionary setting. We found that NoAz is missing between 56 to 159 orthologs that were present in this free-living ancestor. Most of these belong to the functional classes Energy production (10.4%), Inorganic ion transport and metabolism (9.4%), Nucleotide transport and metabolism (9.3%) and Carbohydrate transport and metabolism (9.1%). Numerous orthologs (28 in total) with similarity to ABC-transporters, permeases and secretory proteins were also missing in NoAz, as was a cell division control protein with a DNA helicase domain, known to resolve Holliday junctions that arise during recombination and repair. In contrast, the functional classes Transcription, Defence mechanisms and Translation were the least represented among the missing orthologs.

## Discussion

Our comparative analyses of cyanobacterial genomes revealed a core gene set of 404 orthologs, the majority of which are involved in crucial house-keeping functions. The 58 cyanobacterial genomes examined here provide a dataset several-fold larger than the 15 [17] and 13 [18] genomes analysed in two previous studies. By matching our core orthologs to those identified in these studies, we find that the 1054 cyanobacterial clusters of orthologous groups (CyOGs) identified by Mulkidjanian and co-workers (2006) translate into 1435 of the orthologs in the 58 genomes examined here. The latter study identified a core set of 682 single-copy genes [18], of which 356 are present in our relaxed core (RCF) of 393 single-copy orthologs. The differences noted obviously relate to the number of genomes examined but also to the method used to cluster orthologs and define the core. The method (OrthoMCL) used here to define the core gene set in the 58 genomes involves an all-against-all BLAST step, and additional rules to identify recent paralogs (so called in-paralogs). Mulkidjanian and co-workers (2006) used the cluster of orthologous groups method [46,47] and defined the core in their dataset as CyOGs that were missing in no more than one genome. Of the 1435 orthologs in our dataset, which correspond to the 1054 CyOGs identified by Mulkidjanian and co-workers, 931 are likewise present in our relaxed core gene set (i.e. in either the strict core, RCF or RCF1). The 504 CyOGs missing from the relaxed cores were mostly lacking in cyanobacterium UCYN-A, the two Yellowstone species or in the *Prochlorococcus *clade and belonged primarily to the General prediction only (R), Energy production (C), Amino acid (E) and Inorganic ion transport and metabolism (P) functional categories. Shi and Falkowski [18], on the other hand, identified their 682 single-copy orthologs using reciprocal best BLAST hits and divided these into 323 core and 329 shell genes (the latter displaying divergent phylogenies). The orthologs which are not present among the 393 single-copy genes in our RCF gene set most often corresponded to genes involved in coenzyme and amino acid transport and metbolism. The reason that these orthologs are missing from the core in our dataset is due to their absence in one or more finished genome as well as to the existence of multiple gene copies in one or more of the genomes analysed here.

The phylogeny presented in Figure 1 shows two major cyanobacterial clades and a sister group consisting of the Yellowstone species and *Gloeobacter violaceus*, in agreement with Gupta and Mathews (2010) [19]. Cyanobacteria with the largest genomes (Clade 1) contained the highest number of paralogs. This result suggests that gene duplication is a strong driving force for broadening the phenotypes and subsequently the adaptive behaviour of the cyanobacteria investigated, as proposed for other prokaryotes [27,48-50]. These duplications can originate from either within the genome itself (paralogs) or can be introduced by horizontal gene transfer (HGT). Although we did not attempt to discriminate between these two mechanisms, we observed that approximately 10% of protein groups involved only a single genome (i.e. do not show significant similarity to any protein in the other genomes). This indicates that several protein families within cyanobacteria may be the result of HGT events or that these sequences have diverged to the point where any significant similarity to homologs in other genomes is lost. Additionally, gene transfers both within cyanobacteria and from other phyla have been reported previously [51-53] and may contribute substantially to the expansion of gene families [54]. A striking example of gene family expansion is obvious in the chlorophyll d-containing unicellular *Acaryochloris marina *with the second largest genome among the 58 genomes examined and by far the highest number of paralogs (Figure 2). Indeed, this cyanobacterium is capable of adapting to a range of specialized environmental niches [9], including symbiosis with ascidians [44] and marine macroalgae [55]. The other extreme is the unicellular cyanobacterium UCYN-A with the smallest genome and the lowest number of paralogs among the 58 cyanobacteria investigated. The paralogs identified in this genome appear biased towards posttranslational modifications and chaperone functions (Figure 2), events of importance in endosymbionts [56] and exhibiting a positive selection bias in e.g. the insect endosymbiont *Buchnera *[57]. The drastically diminished genomic features in Ucyn therefore support an implied life-style dependence on other organisms [12,22], potentially in a fully developed endosymbiotic context.

According to our parsimony reconstructions (based on genome sizes and the presence/absence of orthologs in genomes), the genome size of the most recent common ancestor (MRCA) of cyanobacteria was approximately 4.5 Mbp and contained 1678-3291 protein-coding genes. The coding nucleotide proportion would then account for 36-70% of the proposed genome size of the MRCA. Including the number of genes within orthologous groups in the reconstruction (i.e. gene copy number within protein families) did not significantly alter the size of the estimated gene set. The relatively low coding proportion may be due to the fact that estimates are based on orthologs in genomes, thereby missing the genes that could not be grouped by our gene clustering method. A parsimony reconstruction of the total number of genes in the MRCA puts the gene count at approximately 4300, which represents a more plausible size of the ancestral gene repertoire. Interestingly, the MRCA genome may have included a set of *nif *genes, opening up the possibility that the cyanobacterial ancestor was a diazotroph (shown also in Figure 7B).

It is apparent that the genomes of cyanobacteria, ranging almost 10-fold in size from just above 1 Mbp to close to 10 Mbp, have been subjected to a range of niche-specific environmental and competitive forces during the billion years of evolution of the phylum, and thus each genome in our dataset may uniquely reflect the characteristics it displays. Still, the cyanobacterial genome sizes show two general trends that reflect the two major clades identified (Clade 1 and 2; Figure 1). The evolutionary history of genomes within Clade 1 involves a mix of expansions and reductions while a single event of genome reduction appears to have taken place at the branching of Clade 2, dominated by the often closely related marine picocyanobacteria (Figure 4). It is also clear that both genome sizes and number of in-paralogs evolve at a faster rate (Figure 5A,B), and that there exists a strong correlation between genome sizes and gene copy number, in genomes of Clade 1 (Figure 5C). Thus, Clade 1 cyanobacteria appear to employ a survival strategy anchored in genome (and consequently gene family) expansion capacities, while the unicellular and mostly marine cyanobacteria in Clade 2 rely on genome streamlining and the maintenance of a minimal gene repertoire. The former strategy allows Clade 1 cyanobacteria to be highly flexible in terms of not only developmental capacities (e.g. specialization via cell differentiation: akinetes, heterocysts, diazocytes), but also physiological performance (e.g. nitrogen fixation, symbiosis) and a wide habitat occupancy among prokaryotes. A similar pattern has been shown previously within Clade2 cyanobacteria with significant streamlining having ocurred in *Prochlorococcus *isolates since their divergence from *Synechococcus *[58] (the latter considered to be better generalists than the former). In spite of the constraints imposed by the genomic streamlining in Clade 2 cyanobacteria, this strategy has been ecologically highly successful, at least in specific niches, considering the abundance of these cyanobacteria in nutrient-deficient warmer oceans with few competitors, or as recently suggested, positive interactions with other co-occurring bacteria [59,60].

The adaptive potential of the Clade 1 strategy may also explain the successful entrapment in highly specialized niches. A clear example is the obligate symbiont '*Nostoc azollae*' 0708, which has substituted a free-living life-style with one restricted within a eukaryotic host. Still, this symbiotic interaction is highly dependent on the complex developmental modes of the cyanobacterium, involving differentiation of several cell types, including those required for the vertical transmission process [14]. Such host-restricted conditions impose population size diminishments and relaxed constraints on protein-coding genes, leading to fixation of deleterious mutations [61] and pseudogenization followed by gene loss and genome shrinkage [62]. The '*Nostoc azollae*' 0708 is apparently at an early stage of a host-adapted life-style, as its genome size has been only slightly reduced since divergence from its free-living ancestor (5.65 to 5.49 Mbp; Figure 4). This is also reflected in the relatively low number of orthologs (56-159), which we estimate to have been completely lost during symbiosis and the still evident high proportion of pseudogenes [8], which are likely targets for removal. If these pseudogenes were to be lost, the genome size of '*Nostoc azollae*' 0708 would approach that of its closest relatives *C. raciborskii *and *R. brookii *(Figure 3). Whether any of the lost genes have been targeted to the plant nucleus (as in the case of chloroplasts) is unknown. Such a transfer of genetic material from the cyanobiont to the host might be mediated by the DNA-containing vesicles seen during the vertical transmission of the cyanobiont between *Azolla *generations [14].

Other cyanobacteria in Clade 1 that show signs of genome reduction include *Cylindrospermopsis raciborskii *and *Raphidiopsis brookii *[16], the unicellular cyanobacterium UCYN-A [12] and *Thermosynechococcus elongatus *[63] which occupies hot-springs [64]. However in these cases, the genome reduction has affected distinctly different cellular processes. In *Cylindrospermopsis *and *Raphidiopsis*, free-living aquatic cyanobacteria with the smallest genomes (3-4 Mbp) among Section IV heterocystous cyanobacteria, the capacity to differentiate heterocysts has been negatively affected, while in the marine nitrogen-fixing Ucyn, genes involved in photosynthesis (PSII) are lost. The reduction in genome size in these cyanobacteria has been considerably more dramatic than in '*Nostoc azollae*' 0708, since divergence from their last known ancestor (5.65 to < 4 Mbp in Cylr and Rapb; 5.15 to 1.44 Mbp in Ucyn). These genome reductions suggest a symbiotic or other highly interactive life style for these organisms. If this is the case, the former would act as photosynthetic symbionts (e.g. in a heterotrophic host) while Ucyn would act as a diazotrophic symbiont delivering nitrogen to a potentially photosynthetic host supplying carbohydrates. The genome of *Trichodesmium erythraeum *provides another unexpected example in this context as it displays the highest proportion of non-coding nucleotides (including also numerous pseudogenes) among all 58 genomes examined (Figure 3). This implies a severe ongoing genome erosion (in coding functions) in a globally wide-spread, free-living, and fully photosynthetic and diazotrophic cyanobacterium, sharing oligotrophic surface waters with cyanobacteria with the smallest genomes known (e.g. *Prochlorococcus marinus *MIT9313 and cyanobacterium UCYN-A). However, our data also clearly show that the large genome of *Trichodesmium erythraeum *(7.75 Mbp) is not shrinking but expanding in size (Figure 4). This result suggests that this cyanobacterium employs yet another strategy to cover large segments of our oceans. One hypothesis is that the large *Trichodesmium *colonies, which are associated with numerous other microorganisms [65], are highly flexible and 'open' to gene gain via transfers from its co-habitants.

Considering the large number of genomes used in this study, the finding of orthologs that are exclusively shared by, or missing from, genomes with specific phenotypes strongly implicates these orthologs in the underlying reason for the resulting phenotype. For instance, we identified 17 orthologs underpinning filament formation, 96 orthologs exclusive to heterocystous species, as well as 13 orthologs shared between the only two sequenced plant symbionts. Since no orthologs were identified exclusively in the unicellular cyanobacteria, the filamentous phenotype is most likely a derived trait. Our results suggest that the cyanobacterial ancestor was unicellular and that the filamentous morphology was introduced later within the lineage (Figure 7A). This finding is in line with previous reports on cyanobacteria [36,37] and argues against the irreversible end-state of the coccus morphology in prokaryotes [66].

Not less than 21 of the 96 heterocyst-related orthologs are pseudogenes in '*Nostoc azollae' *0708, suggesting that they are not directly involved in heterocyst formation or that heterocysts are in some way severely impaired in NoAz. Among the heterocystous cyanobacteria, the N-terminal PATAN domain of PatA [42] is lacking in *Cylindrospermopsis raciborskii *and the whole *patA *gene is missing in *Raphidiopsis brookii*. The absence of a full-length copy of this otherwise heterocyst-specific ortholog is a likely reason for the exclusively terminal heterocysts (which in *Raphidiopsis *are not fully developed) in these strains.

As seven of the symbiont-specific genes are no longer functional in the obligate '*Nostoc azollae*' 0708, these may in turn have key functions in the re-establishment of plant-cyanobacterial associations. The inability of '*Nostoc azollae*' 0708 to survive outside the plant host has puzzled researchers. The cyanobacterial symbiont of *Azolla *could be kept alive for months under certain free-living conditions, but was still unable to multiply and exhibited photobleaching [15]. Our finding of pseudogenized orthologs in NoAz involved in pigment synthesis and present in all other cyanobacterial genomes investigated, suggests that these genes are necessary for survival under free-living conditions but dispensable in the *Azolla *symbiosis where the chloroplast canopy of the plant shields the symbiont from excess light. Moreover, the discovery of a specific protein family related to carbohydrate metabolism in the two plant symbionts *Nostoc punctiforme *and '*Nostoc azollae*' 0708, makes the substrates of this enzyme, N-acetyl-D-glucosamine and D-glucosamine, a likely candidate for the carbohydrate supplied by cyanobacterial plant hosts. Interestingly, D-glucosamine is a signalling factor for nodule development in *Rhizobia *symbioses [67]. These orthologs constitute exciting targets for further analyses.

Finally, we are aware that bacterial traits often require a complex network of genetic interactions and gene expression dosage, seldom explained by the presence or absence of a single gene product. Furthermore, the comparative approach taken here is naturally limited by the data (sequenced genomes) at hand. Importantly, the organisms included should optimally be judiciously sampled in order to reflect the underlying phylogenetic diversity and genetic complexity. Such a representative sample might be hard to achieve given the current availability of cyanobacterial genomes, but should definitely be taken into account in further sequencing efforts. Nevertheless, we hypothesize that linking protein families to characteristics, and analysed from an evolutionary perspective, provides a valuable starting point for further research on the metabolic and morphological phenotypes of cyanobacteria.

## Conclusions

With the dataset used, based on 58 cyanobacterial genomes, the stringent core genetic repertoire of cyanobacteria now represents 404 orthologs. A relaxed core gene set, based on 39 of these genomes sequenced to completion, contains 536 orthologs, of which 393 are found in single-copy versions. Due to the severe loss of genes in cyanobacterium UCYN-A neither of these core gene sets include genes for the photosystem II complex (although these are present among 268 additional orthologs allowed to be missing from one finished genome). Two orthologs in the stringent gene core are unique to all cyanobacteria, one which corresponds to a transcriptional regulator and the other to an unknown protein with a conserved genomic neighbourhood in all species examined. Seven additional orthologs were unique to the relaxed core gene sets of the cyanobacterial phylum (allowed to be missing from 1-3 of the finished genomes) but are currently poorly characterized.

Genome sizes and number of duplicated genes within the major clade, which comprises species of varying complexity and choice of habitat (including marine and freshwater, unicellular and filamentous and heterocystous species) evolve at a higher evolutionary rate than the clade of marine picocyanobacteria. Variation in cyanobacterial genome sizes is the result of a mix of gains of losses in the former clade and of one single reduction event in the latter. It is also deduced that the common ancestor of extant cyanobacteria had a genome size of ~4.5 Mbp and contained between 1678 and 3291 proteins, ~4-6% of which are unique to cyanobacteria today. The largest genomes of cyanobacteria contain the highest number of paralogs and there is a strong correlation between genome and gene family expansions within the more complex clade. The latter is likely to result in increased adaptive potential but may also lead to a an accumulation of non-coding genomic regions as the organisms shift their habitat and/or life-style, rendering duplicated genes redundant and subject to increased mutation rates. A clear example of this phenomenon is the obligate symbiont '*Nostoc azollae*' 0708 in which 64% of in-paralogs are pseudogenes.

A number of orthologous proteins underpin traits of certain cyanobacterial groups. These include a set of signal transduction proteins and a glycosyl hydrolase specific to plant symbionts. Conversely, the lack of certain conserved orthologs or domains in a few cyanobacteria can be correlated with the phenotype they exhibit as seen in *Cylindrospermopsis raciborskii *with terminal heterocysts only, and the loss of functional genes involved in pigment biosynthesis in '*Nostoc azollae*' 0708.

## Methods

### Dataset

All available cyanobacterial genomes as of June 24th 2010 were downloaded from NCBIs genome database FTP server. The dataset included 58 genomes in total, 39 of which were sequenced to completion. Sequences for all protein coding open reading frames (ORFs) and predicted pseudogenes, the latter acquired from the IMG database [68], were included in the analysis.

### Orthologous protein groups and annotations

All ORFs in the dataset were subjected to orthologous protein grouping using OrthoMCL v.2.0.1 [69,70]. Protein sequences for each group were aligned with MUSCLE v.3.5.1 [71], and Hidden Markov Models (HMMs) [72] were built from the alignments using hmmbuild v.3.0 [73]. Pfam and Uniprot databases (26 August 2010) were searched with the HMMs as queries using hmmsearch v.3.0 [73] and annotations were assigned to orthologous groups for hits with full and domain-specific e-values < 0.01 and a bias/score ratio < 10. COG functional groups were assigned to orthologous groups using RPS-BLAST against the CDD database (23 August 2010) with all sequences of a group as query. For each group, the most predominant best hit among orthologous sequences (at e-value cut-off 0.01) was assigned. In case the majority of sequences within a group could not be assigned a COG hit, the second most abundant COG (if available) was assigned to the group. Proteins which were not clustered by OrthoMCL were assigned the highest scoring COG at e-value threshold < 0.01. Cyanobacteria-unique protein groups were defined as those which contained proteins with only cyanobacterial hits below an e-value threshold of 0.01 in the nr database.

### Phylogenetic analysis

A phylogenetic analysis was conducted on a set of 285 orthologous protein groups (see additional file 2) in the output from OrthoMCL (see above) present in single-copy number in all 58 genomes in the dataset. Proteins in each orthologous group were aligned using MUSCLE (with default settings) and concatenated in to one supermatrix of 128,769 amino acid positions (see additional file 8). Amino acid substitution models for each individual group was selected by the Akaike Information Criterion (AIC) obtained from ProtTest v.3.0 [74] which uses PhyML v.3.0 [75] for likelihood calculations. The amino acid substitution models implemented both in PhyML and RaxML (see below) were used as candidate models for the AIC ranking. The 285 groups were analysed separately and combined under maximum likelihood as implemented in RAxML v.7.2.6 [76] and clade support was assessed using bootstrapping [77]. For the separate analyses, the AIC-best models were used in RAxML. To avoid overparameterisation [78] but still allow for evolutionary heterogeneity, the concatenated alignment were analysed using a mix-model approach where each gene partition was lumped in a "substitution model"-partition according to their AIC-best chosen models. This yielded a five partition model ("LG", "JTT", "CPREV", "WAG", "RTREV") each combined with an assumption of a rate heterogeneity across sites modelled as a discrete Gamma distribution.

Shortcomings of the models used in phylogenetics can render tree inference of prokaryotic organisms problematic [79] (or even questionable [80]), and the presence of phylogenetic incongruence in our data was assessed using a SH-test [81,82] as implemented in RAxML. The trees from the separate gene analyses were pooled together with the tree from the concatenated supermatrix (τ_285_), and the SH test was run for each gene data. The separate gene analyses yielded a large collection of trees, thus suggesting extensive topological incongruence (see additional file 9). We did not investigate whether lateral gene transfer could be a source of incongruence but made the assumption that we may estimate one underlying tree structure for our data sample (cf. [80]). Thus, we note that concatenating and analysing the set of genes (N = 71) for which τ_285 _could not be rejected, gave a tree (τ_71_) very similar to the tree resulting in analysing all 285 genes (τ_285_). The two trees differed only in two, poorly supported nodes (bootstrap proportions < 60%), and we chose to base our analysis on the 285-gene tree. Trees were rooted on the *Gloeobacter violaceus *branch, following previous results as having *G. violaceus *as the sister group to all extant cyanobacteria e.g., [19,37,83,84].

### Genome composition

Characters of the cyanobacterial genomes, such as sizes and presence of orthologous groups were analysed by parsimony optimization (least squared for continuous characters) over the phylogenetic tree using Mesquite (version 2.74) [85]. Analysis of evolutionary rates of genome sizes and paralogs was performed using Felsenstein's phylogenetically independent contrasts [31] as implemented in the PDAP module [86] of Mesquite.

## Competing interests

The authors declare that they have no competing interests.

## Authors' contributions

JL and JAAN carried out the genomic analyses, participated in its design and drafted the manuscript. BB coordinated the study, participated in its design and drafted the manuscript. All authors read and approved the final manuscript.

## Supplementary Material

Additional file 1 **Overview of the 58 cyanobacteria which genomes were examined in this study**. Information on general genome features and characteristics of species is provided. Click here for file

Additional file 2 **Orthologs in the cyanobacterial core**. List of orthologs present in all genomes examined. Gene locus tags and functional annotations (for the Uniprot, Pfam and COG databases) are provided Click here for file

Additional file 3 **Orthologs missing in finished genomes**. List of orthologs which are missing, or pseudogenized, in finished genomes while present in all others genomes. Locus tags for pseudogenes and functional annotations are provided. Click here for file

Additional file 4 **Orthologs present in the most recent common cyanobacterial ancestor**. List of orthologs predicted in the most recent common ancestor of cyanobacteria. Functional annotations and gene locus tags in each genome (when available) is provided. Click here for file

Additional file 5 **Orthologs specific to filamentous cyanobacteria**. List of orthologs present in all filamentous cyanobacteria with finished genomes and missing from all unicellular cyanobacteria. Click here for file

Additional file 6 **Orthologs specific to heterocystous cyanobacteria**. As above. Click here for file

Additional file 7 **Orthologs specific to plant-symbiotic cyanobacteria**. As above. Click here for file

Additional file 8 **Individual and concatenated alignments for protein groups**. Compressed file containing all alignments of individual groups (fasta format) and the concatenated alignment of 285 single-copy genes (phylip format). Click here for file

Additional file 9 **Details on test of incongruence**. Description of statistical analyses carried out on phylogenetic trees from the 285 single-copy orthologs and the concatenated alignment. Click here for file
