# Supplementary material for: Genome fluctuations in cyanobacteria reflect evolutionary, developmental and adaptive traits
Source: BMC Evol Biol. 2011 Jun 30;11:187. doi: 10.1186/1471-2148-11-187 (PMC3141437; doi:10.1186/1471-2148-11-187)
Supplement: Additional file 9 — Details on test of incongruence. Description of statistical analyses carried out on phylogenetic trees from the 285 single-copy orthologs and the concatenated alignment. [file 1471-2148-11-187-S9.PDF]

Genome Fluctuations in Cyanobacteria Reflect Evolutionary, Developmental and Adaptive Traits: Details on Tests of Incongruence

J. Larsson, J.A.A. Nylander, B. Bergman

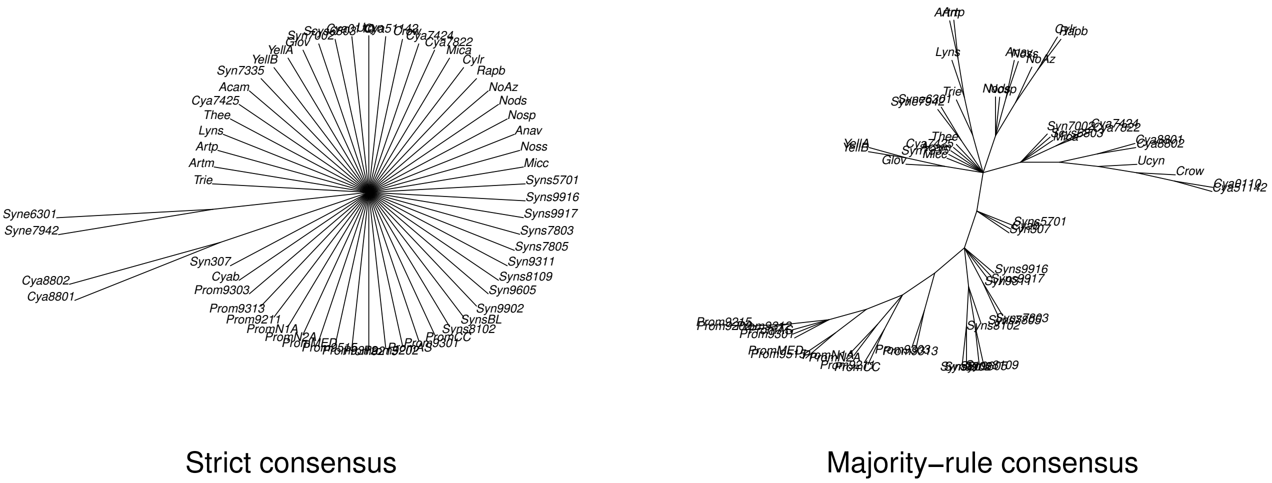

Figure 1: Separate maximum likelihood analyses of the orthologous groups (see main text for details) yielded 285 trees with unique topologies. This is a remarkable finding in that all genes gave different tree estimates. The strict consensus was virtually unresolved – indicative of potential extensive incongruence, while a majority-rule consensus revealed some common structure.

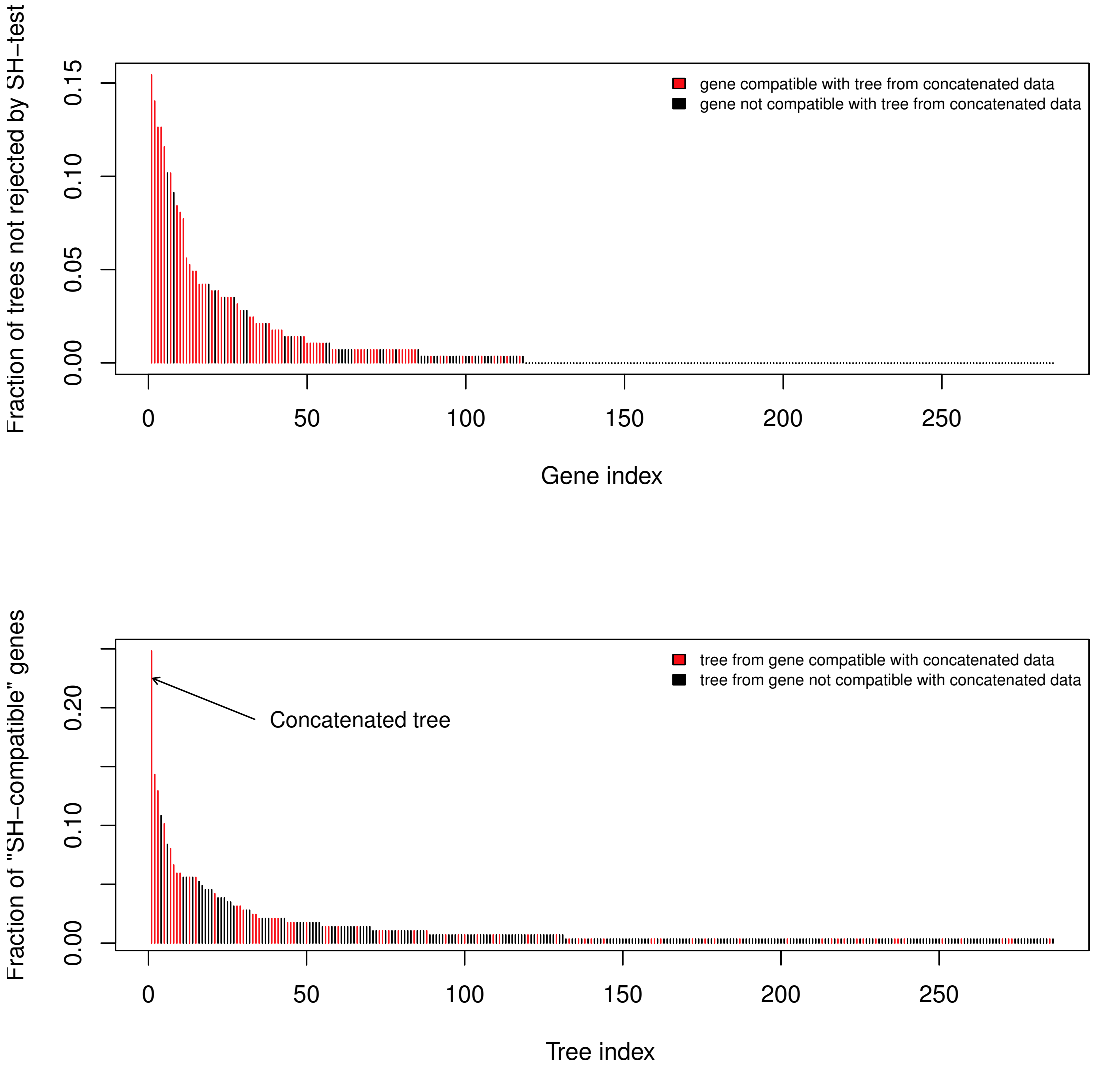

Figure 2: A SH-test [1][2] as implemented in RAxML v.7.2.6 [3] was carried out for each individual gene on the pooled sample of trees resulting from the individual analyses, as well as a ML tree from the concatenation of all genes (ML_{Concat}). Total number of trees was 286.

The upper panel show the fraction of trees not rejected in the SH-test per gene. Genes are ordered on the x-axis in descending (y-value) order. Red colored bars indicate those genes which did not reject ML_{Concat}. A high fraction indicates that the the particular gene fails to reject a higher number of trees in the SH-test. Slightly more than half of the genes rejects a maximum fraction of trees (rejecting all other trees than the ML tree inferred from the gene it self), indicating that those genes have a strong conflicting signal compared to the rest. The remaining genes are slightly more ambiguous in their point estimates, and the distribution of red-coloring show that the ML_{Concat} could not be rejected for most of them. It is worth noting that several of the genes in the right-end of the scale (Gene index around 90–120), only fails to reject their ML estimate, and ML_{Concat}, respectively.

The lower panel show the fraction of genes that does not reject a particular tree in the SH-test. Trees are ordered on the x-axis in descending (y-value) order. The tree from the concatenated data set is indicated with an arrow. Red colored bars indicate those trees derived from data compatible with the concatenated data. A high fraction indicates a particular tree topology compatible with a larger number of genes. Not surprisingly, the ML_{Concat} is the least refuted, being "not significalty worse" as an explanation for 25% (N=71) of the genes. Again, the distribution of the red-coloring of the bars reveals that many of the trees are from genes which could not reject the ML_{Concat}.

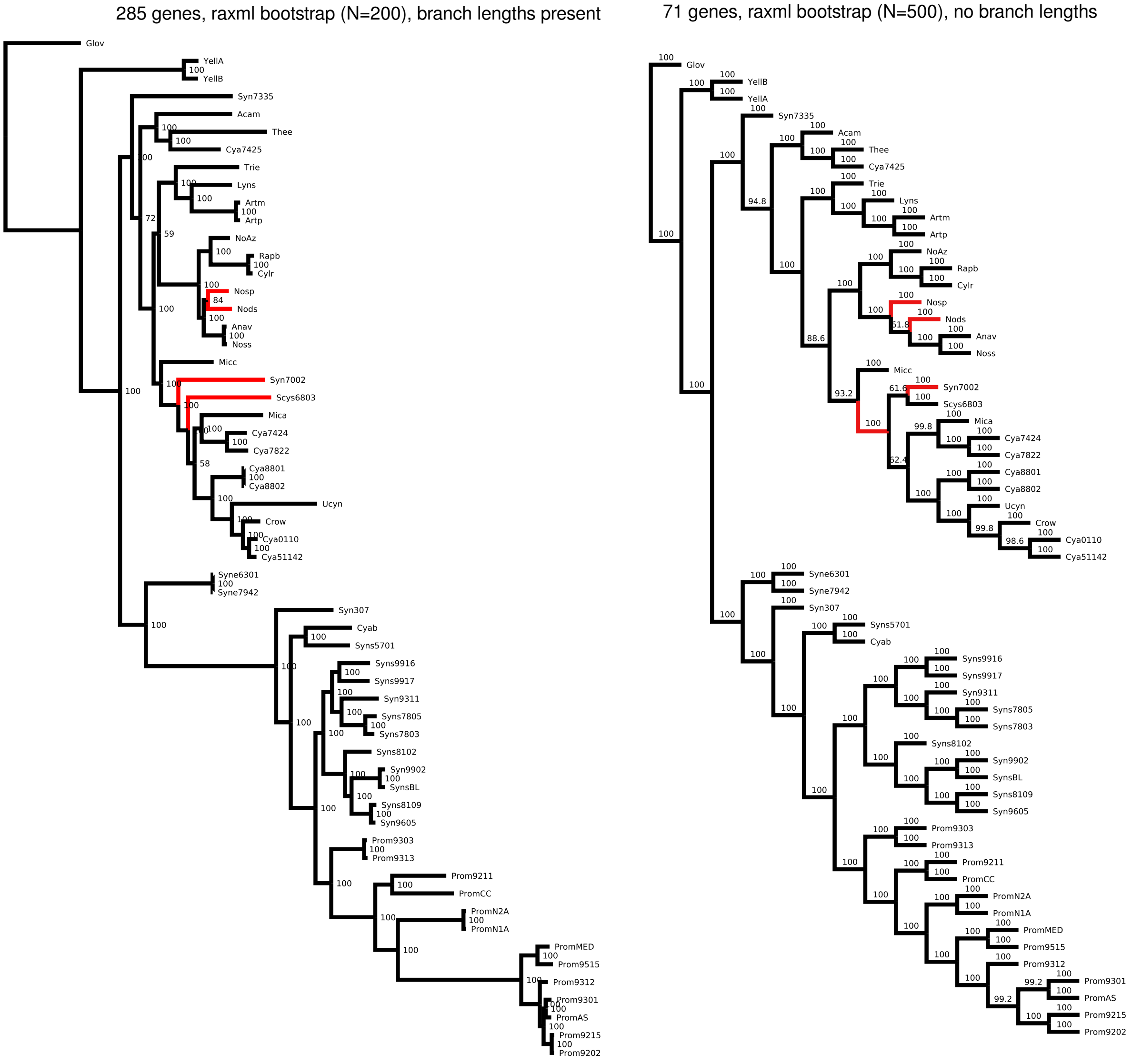

Figure 3: A comparison of the ML tree from a RAxML analysis of the concatenated 285 gene data (ML_{Concat}), and an analysis of a concatenation of 71 genes found "compatible" with ML_{Concat} in the SH-test. The ML_{Concat} (to the left) and the 71-gene tree (to the right) differ only in two groupings, both involving low bootstrap support (∼60%). Red color indicates branches affected.

Bibliography

[1] H. Shimodaira and M. Hasegawa. Multiple comparisons of log-likelihoods with applications to phylogenetic inference. *Mol. Biol. Evol.*, 16:1114–1116, 1999.

[2] Hidetoshi Shimodaira. An approximately unbiased test of phylogenetic tree selection. *Syst. Biol.*, 51:492–508, 2002.

[3] Alexandros Stamatakis. RAxML-VI-HPC: Maximum likelihood-based phylogenetic analyses with thousands of taxa and mixed models. *Bioinformatics*, 22:2688–2690, 2006.
